# E- Cigarette acute effect on symptoms and airway inflammation: comparison of nicotine with a non-nicotine cigarette

**DOI:** 10.1186/1617-9625-12-S1-A35

**Published:** 2014-06-06

**Authors:** Sophia Vakali, Stamatoula Tsikrika, Sophia Antiopi Gennimata, Georgios Kaltsakas, Anastasios Palamidas, Nikolaos Koulouris, Christina Gratziou

**Affiliations:** 1 Research unit for Tobacco Control, 1st Respiratory Department, University of Athens, Medical School, Sotiria Hospital, Athens, 11527, Greece

## Background

Despite the increasing advertising of e-cigarettes as safe smoking tool, there is much debate regarding its safety. This study was undertaken to assess the effect of a single e-cigarette use on clinical symptoms, vital signs and airway inflammatory markers after inhaling either 0mg or 11mg of nicotine.

## Materials and methods

We studied 64 subjects (aged 22-65 years, 34 men) divided in 2 groups. Group A: 12 never smokers and 29 healthy smokers smoked for 10 min a single e-cigarette containing 11mg of nicotine and Group B: 14 never smokers and 9 healthy smokers smoked a single e-cigarette containing 0mg of nicotine. The same brand of e-cig was used in both groups with similar liquid `ingredients but with two different nicotine concentrations. Vital signs, symptoms questionnaire, Oxygen Saturation (SpO2) ,heart rate(HR)] and indices of airway inflammation(exhaled NO, and airways temperature) were assessed pre and post smoking.

## Results

All subjects reported symptoms immediately after smoking, but the respiratory (sore throat, cough) and the cardiovascular symptoms (palpitations) were reported more often in Group A compared with Group B, whereas dizziness, was more frequently reported from non smokers of Group B. An increase in HR was noted in all subjects of Group A, findings that were not recorded in group B. A decrease in FeNO was detected in smokers and non-smokers of Group B, with an increase in airways temperature (p=0.051) in smokers of Group A.

## Conclusions

Increased heart rate, palpitations and a decrease in SpO2 , are related to the use of a nicotine containing e-cig but airways symptoms (sore throat, cough) and inflammatory markers are independent of nicotine use.

